# Palladium-Catalyzed Modification of Unprotected Nucleosides, Nucleotides, and Oligonucleotides

**DOI:** 10.3390/molecules20059419

**Published:** 2015-05-22

**Authors:** Kevin H. Shaughnessy

**Affiliations:** Department of Chemistry, The University of Alabama, Box 870336, Tuscaloosa, AL 35487-0336, USA; E-Mail: kshaughn@ua.edu; Tel.: +1-205-348-4435; Fax: +1-205-348-9104

**Keywords:** nucleosides, nucleotides, oligonucleotides, palladium, cross-coupling, aqueous-phase catalysis

## Abstract

Synthetic modification of nucleoside structures provides access to molecules of interest as pharmaceuticals, biochemical probes, and models to study diseases. Covalent modification of the purine and pyrimidine bases is an important strategy for the synthesis of these adducts. Palladium-catalyzed cross-coupling is a powerful method to attach groups to the base heterocycles through the formation of new carbon-carbon and carbon-heteroatom bonds. In this review, approaches to palladium-catalyzed modification of unprotected nucleosides, nucleotides, and oligonucleotides are reviewed. Polar reaction media, such as water or polar aprotic solvents, allow reactions to be performed directly on the hydrophilic nucleosides and nucleotides without the need to use protecting groups. Homogeneous aqueous-phase coupling reactions catalyzed by palladium complexes of water-soluble ligands provide a general approach to the synthesis of modified nucleosides, nucleotides, and oligonucleotides.

## 1. Introduction

**Figure 1:**
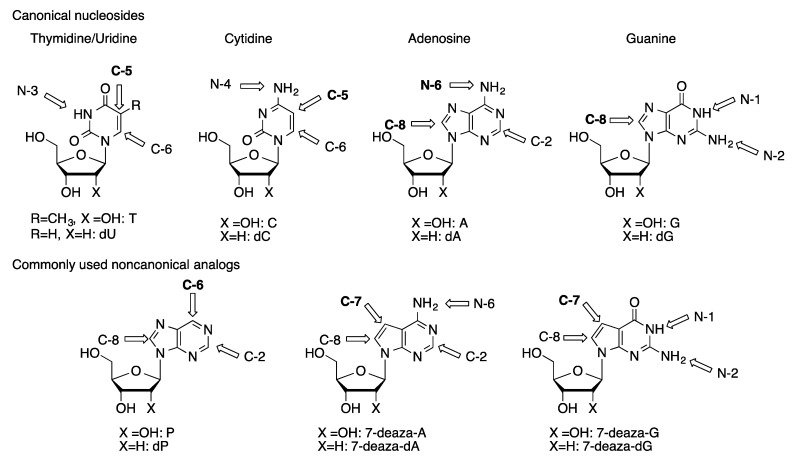
Typical functionalization sites in naturally occurring nucleosides and commonly used unnatural analogs. Most common functionalization sites are bolded.

Nucleosides are one of the fundamental building blocks in biochemistry. There has been long-standing interest in the synthesis of non-natural analogs of nucleosides. The modified nucleosides, or their nucleotide or oligonucleotide analogs, have been widely explored as pharmaceutically active compounds [1,2,3,4], in the study of carcinogenesis mechanisms [5,6,7], and to incorporate probe functionality in DNA and RNA [8,9,10,11]. Nucleoside derivatives can be made through manipulation of the sugar moiety, replacement of the purine or pyrimidine heterocycle, or through covalent modification of the natural heterocyclic base or their analogs. Functionalization of the canonical nucleoside scaffolds and their close derivatives (Figure 1) provides a general approach to the synthesis of base-modified nucleosides [12,13,14].

Metal-catalyzed cross-coupling reactions are powerful methods to form C-C and C-heteroatom bonds with aromatic and heteroaromatic structures [15]. These reactions can often be carried out under mild conditions and can be highly functional group tolerant. Palladium-catalyzed cross-coupling reactions have been widely used in the synthesis of base-modified nucleoside derivatives [16,17,18,19,20,21,22]. Halogenated nucleoside derivatives can be prepared by standard electrophilic aromatic halogenation reactions. The halonucleosides can be coupled with a wide variety of nucleophilic reagents to form carbon-carbon or carbon-heteroatom bonds (Figure 1). Amine-moieties on nucleoside rings can also be arylated. The rapid development of direct arylation methodologies has resulted in the reports of several methods for the direct arylation of C-H bonds on nucleoside bases.

**Scheme 1:**
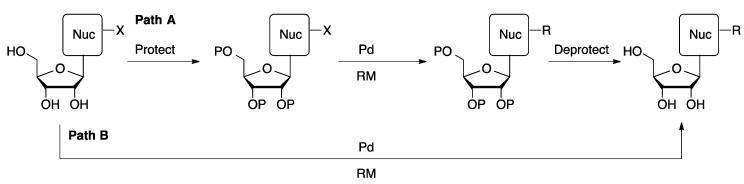
Synthetic approaches to palladium-catalyzed nucleoside modification.

Although palladium-catalyzed cross-coupling reactions are well precedented for a wide range of substrates, nucleosides present a number of challenges. Heterocycles are often challenging substrates in cross-coupling reactions because of their ability to coordinate to metal catalysts and deactivate them. In addition, heterocycles are often electronically very different from more typical aromatic substrates, which can hinder steps in the catalytic cycle. For example, electron-rich halogenated heterocycles, such as furans, thiophenes, and pyrroles, undergo slow oxidative addition to metal complexes. In addition to the challenges of cross-coupling highly functionalized heterocyclic substrates, the polar nature of nucleoside derivatives often results in them being poorly soluble in typical organic solvents. One common approach is to protect the ribose alcohols as esters or silyl ethers to provide a more hydrophobic substrate (Scheme 1, Path A). This approach introduces two additional synthetic steps, which results in decreased yields and poor atom economy. Protection strategies are typically not effective for the more hydrophilic nucleotides and oligonucleotides.

A more attractive approach would be to use unprotected nucleoside, nucleotide, or oligonucleotide derivatives directly in cross-coupling reactions (Scheme 1, Path B). This can be accomplished using polar organic solvents, such as DMF, in the case of nucleosides. Water represents a more attractive solvent for these types of reactions. Water alone or in combination with organic cosolvents effectively dissolves nucleosides and nucleotide derivatives, which allows reactions to be carried out under homogeneous conditions without the need for protection strategies. In this review, development of methods for functionalization of unprotected nucleosides using palladium-catalyzed cross-coupling in water and polar organic solvents will be reviewed.

## 2. Cross-Coupling of Unprotected Nucleosides with Ligand-Free Palladium Catalysts

**Scheme 2:**
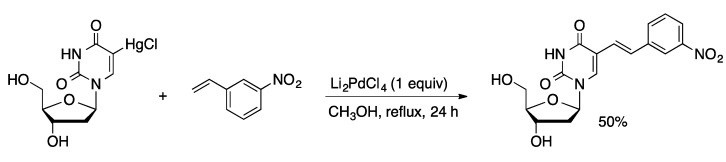
Pd-mediated coupling of 5-HgCldU and styrenes.

**Scheme 3:**
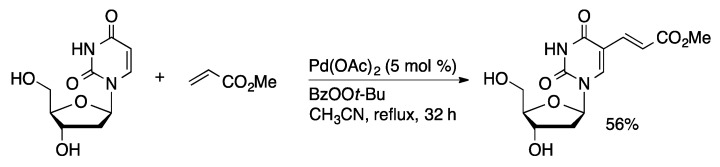
Oxidative coupling of dU and methyl acrylate.

The earliest examples of cross-coupling of unprotected nucleosides were promoted by ligand-free palladium salts in polar organic solvents. Mertes reported a Heck-type coupling of 5-(HgCl)dU with styrene derivatives mediated by stoichiometric Li_2_PdCl_4_ in methanol to give 5-alkenyl-dU compounds (Scheme 2) [23]. The method was also applied to 5-(HgOAc)dUMP. Direct 5-alkenylation of uridine and 2′-deoxyuridine was achieved in modest yield (35%–57%) using catalytic Pd(OAc)_2_ with *t*-butyl perbenzoate in acetonitrile (Scheme 3) [24].

As palladium-catalyzed cross-coupling reactions developed into widely used synthetic methodologies, significant effort has been devoted to developing highly active and general catalyst systems. Much of this effort has focused on developing supporting ligands that increase the reactivity and stability of the palladium center compared to ligand-free palladium catalysts. Ligand free-systems have received renewed attention in recent years because they avoid the use of phosphines or related ligands, which can often be a larger cost in the reaction than the palladium source [25]. Colloidal or nanoparticle palladium catalysts can often provide good levels of activity with aryl iodides and bromides. Recently, ligand-free palladium catalysts have been applied to coupling reactions of unprotected nucleoside substrates.

### 2.1. Cross-Coupling with Organosilanes and Organoboranes

**Scheme 4:**
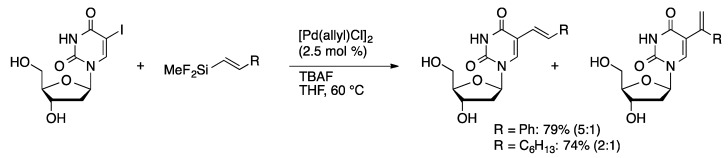
Hiyama coupling of 5-IdU.

Hiyama reported the first examples of a modern cross-coupling of an unprotected halonucleoside with an organometallic reagent. The coupling of 5-IdU with 1-alkenyldifluoromethylsilanes catalyzed by [Pd(allyl)Cl]_2_ was accomplished in THF [26]. A mixture of 1-alkenyl and 2-alkenyl products were obtained (Scheme 4).

**Scheme 5:**
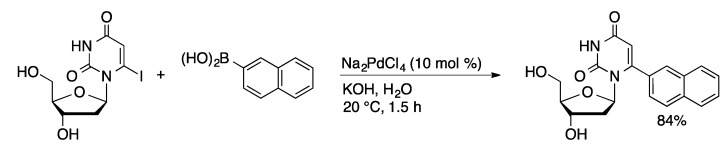
Ligand-free Suzuki coupling of 6-IdU.

Suzuki coupling of 5-IdU with arylboronic acids has been successfully achieved using Na_2_PdCl_4_ without a supporting ligand in water with microwave heating at 100 °C [27,28]. Moderate to excellent yields (33%–85%) were achieved using 0.050–1 mol % palladium with reactions times of one hour or less. The more reactive 6-IU underwent coupling at room temperature with a variety of aryl boronic acids using 10 mol % Na_2_PdCl_4_ (Scheme 5) [29]. To date there are no examples of coupling of less reactive 8-bromopurine nucleosides using palladium without supporting ligands. Ligand-supported catalyst systems are generally required to activate electron-rich aryl bromides, such as 8-bromopurines.

### 2.2. Heck Coupling

**Scheme 6:**
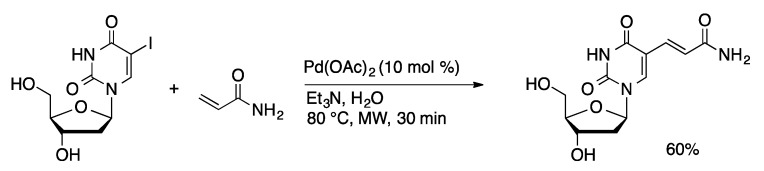
Heck coupling of 5-IdU with a ligand-free palladium catalyst.

Heck couplings catalyzed by palladium without supporting ligands are well precedented [30]. The Heck coupling of 5-IdU and *N*-allyl trifluoroacetamide was achieved in 44% yield at room temperature in water using excess palladium (10 equiv) [31]. Use of commercially available 5-IdU was preferable to previous routes that relied on 5-mercurated uridine. Heck coupling of styrene derivatives and 5-IdU occur in good yield using stoichiometric K_2_PdCl_4_ in DMF at 80 °C [32]. The reaction can be achieved using catalytic amounts of palladium under similar conditions. Heck coupling of 5-IdU with acrylates was achieved in modest to high yield (35%–90%) using Pd(OAc)_2_ (10 mol %) at 80 °C in water under microwave irradiation (Scheme 6) [33].

### 2.3. Direct Arylation via C-H Activation

**Scheme 7:**
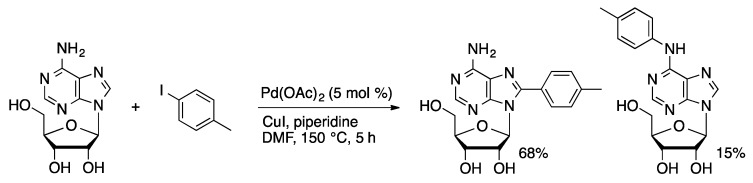
Oxidative coupling of 4-iodotoluene and adenosine.

The direct coupling of heterocycles with arenes has received significant interest in recent years [34,35]. Electron-rich arenes can be coupled with aryl halides under oxidative conditions with high selectivity for the cross-coupled product. The purine nucleosides are effective coupling partners with aryl iodides. 6-(4-Methoxyphenyl)purine ribonucleoside was coupled with aryl iodides in modest yield (27%–50% yield) catalyzed by Pd(OAc)_2_ (5 mol %) using stoichiometric CuI in DMF at 125 °C [36]. The coupling reaction was specific for the electron-rich *C*8-position. The coupling protocol was also applied to adenosine to give a mixture of *C*8- and *N*6-arylated products in 4–5:1 ratios (Scheme 7). Switching the base from piperidine to Cs_2_CO_3_ improved the selectivity for *C*8-arylation of adenosine under otherwise similar conditions [37,38]. The less stable 2′-deoxyadenosine gave significant depurination at 125 °C. Lowering the reaction temperature 80 °C, allowed 8-ArdA derivatives to prepared in 84% yield.

**Scheme 8:**
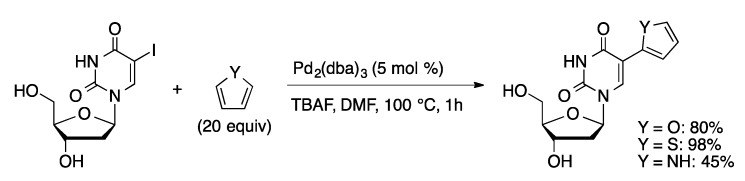
Coupling of 5-IdU and thiophene.

Electron-deficient pyrimidines are more difficult to C-H activate than purines. By reversing the nature of the reaction it is possible to coupling halogenated pyrimidine nucleosides with electron-rich heterocycles. Direct coupling of 5-IU and 5-IdU with furan, thiophene, and pyrrole was achieved using 5 mol % Pd(dba)_2_ in the presence of TBAF in DMF (Scheme 8) [39]. Coupling occurred selectively to give 5-(2-heteroaryl)uridine or 2′-deoxyuridine derivatives.

## 3. Cross-Coupling of Unprotected Nucleosides with Palladium Complexes of Hydrophobic Ligands

Palladium catalysts without supporting ligands are effective catalysts for cross-coupling of iodopyrimidine nucleosides, but have not been effectively applied to coupling reaction of the less reactive 8-halopurine derivatives. Phosphine and *N*-heterocyclic carbene ligands provide more active catalyst systems that can effectively activate all classes of aryl halides. Typical supporting ligands, such as triphenylphosphine, are highly hydrophobic. Using these catalyst species for coupling reactions of unprotected nucleosides requires identifying solvent systems that can solubilize both the hydrophobic catalyst and the hydrophilic nucleoside. Aprotic dipolar solvents, such as DMF and NMP, can be effective solvents for these reactions. Alternatively, mixed aqueous-organic solvent systems can be used effectively. Hydrophobic catalysts can exhibit high activity in reactions run with water as the solvent, even when all reagents are hydrophobic [40]. These "on-water" reactions represent a potential future area of exploration for nucleoside coupling reactions.

### 3.1. Suzuki Couplings

Wagenknecht reported the first example of Suzuki coupling of an unprotected nucleoside [41,42]. 1-Pyrenylboronic acid was coupled with 5-IdU using Pd(PPh_3_)_4_ (10 mol %) in a solvent system composed of THF/MeOH/H_2_O (2:1:2, Scheme 9). The coupled product was isolated in 70% yield. In comparison, coupling of acetyl-protected 5-IdU with 1-pyrenylboronic acid followed by deprotection with NaOMe gave a 55% overall yield. Anthraquinone-labeled uridine was prepared by coupling of 9,10-dimethylanthracen-2-yl pinacolatoborane with 5-IdU catalyzed by Pd(PPh_3_)_4_ (5 mol %) in THF/MeOH/H_2_O followed by oxidation to afford 5-(2-anthraquinonyl)dU in 50% yield over two steps (Scheme 10) [43].

**Scheme 9:**
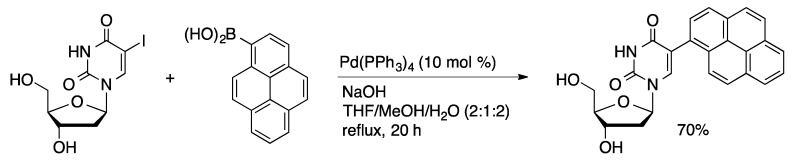
Suzuki coupling of 5-IdU and 1-pyrenylboronic acid catalyzed by Pd(PPh_3_)_4_.

**Scheme 10:**
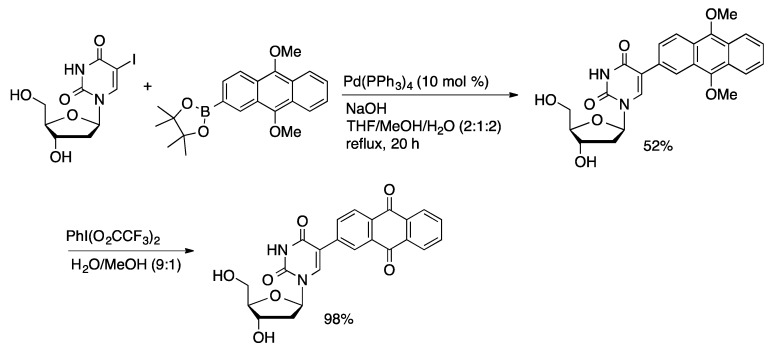
Synthesis of anthraquinone-labeled uridine.

**Scheme 11:**
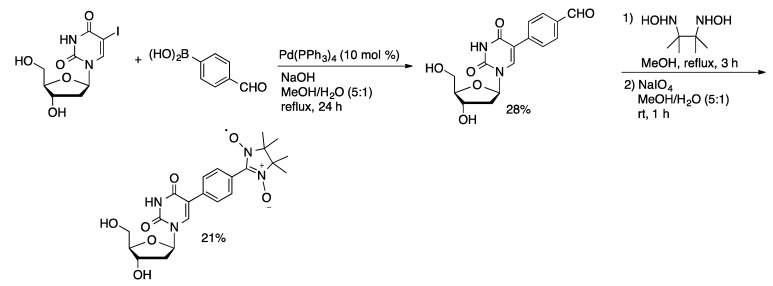
Synthesis of a spin-labeled uridine derivative via a Suzuki coupling.

Other solvent systems have been used in the Pd/PPh_3_-catalyzed Suzuki coupling of unprotected halonucleosides. The synthesis of a novel spin-labeled uridine derivative began with the coupling of 4-formylphenylboronic acid and 5-IdU catalyzed by Pd(PPh_3_)_4_ in methanol/water (Scheme 11) [44]. The 5-aryldU derivative was then converted to 5-(2′-phenyl-4′,4′,5′,5′,-tetramethylimidazoline-3′-oxy-1′-oxyl)dU by a sequence of steps. A protected version of the spin-labeled nucleoside was incorporated into oligonucleotides using the phosphoramidite method and used for EPR analysis of DNA structures. Aqueous DMF was used for the Pd(PPh_3_)_4_-catalyzed coupling of arylboronic acids and 5-IdU [45]. After monophosphorylation, the 5-aryldUMP derivatives were explored as potential anti-tuberculosis agents.

Despite the insolubility of triphenylphosphine in water, Suzuki coupling of arylboronic acids and 5-IdU have been successfully catalyzed by Pd(OAc)_2_/PPh_3_ with water as the only solvent at 120 °C using microwave irradiation [46]. Notably, the PPh_3_-based catalyst provided comparable yields to those obtained using the water-soluble phosphine sodium tri(2,4-dimethyl-5-sulfonatophenyl)phosphine (TXPTS).

**Scheme 12:**
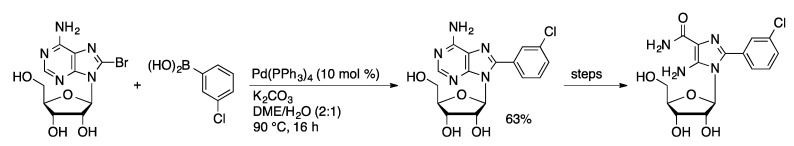
Suzuki coupling of 8-BrdA as first step in AICAR synthesis.

Triphenylphosphine-based catalysts have also been applied to Suzuki arylation of unprotected purine nucleosides. Coupling of polycyclic arylboronic acids with 8-BrdG catalyzed by Pd(PPh_3_)_4_ in THF/MeOH/H_2_O (2:1:2) afforded 8-(1-pyrenyl)dG (65%), 8-(1-pyrenyl)dA (10%), and 8-(6-benzo[*a*]pyrenyl)dG (25%) [47,48]. A family of 8-arylA adducts were prepared using Pd(PPh_3_)_4_ in DME/H_2_O (2:1). The products were then converted to 5-amino-1-β-d-ribofuranosylimidazole-4-carboxamide derivatives (AICAR, Scheme 12), which were explored as potential AMP-activated protein kinase activators [49]. Suzuki coupling of *trans*-β-styrylboronic acid with 8-BrdG catalyzed by Pd(PPh_3_)_4_ in DMF affords 8-(*trans-*styrenyl)dG in 64% yield as a single alkene isomer [50]. The product was used in the study of photochemical *E* to *Z* isomerization of the functionalized guanosine derivative.

**Scheme 13:**
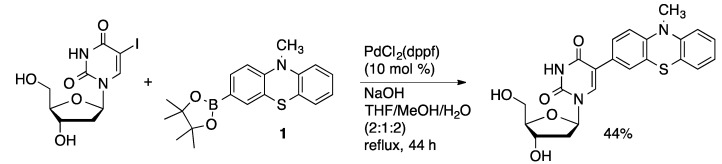
Synthesis of 5-(10-methylphenothiazin-3-yl)dU.

**Scheme 14:**
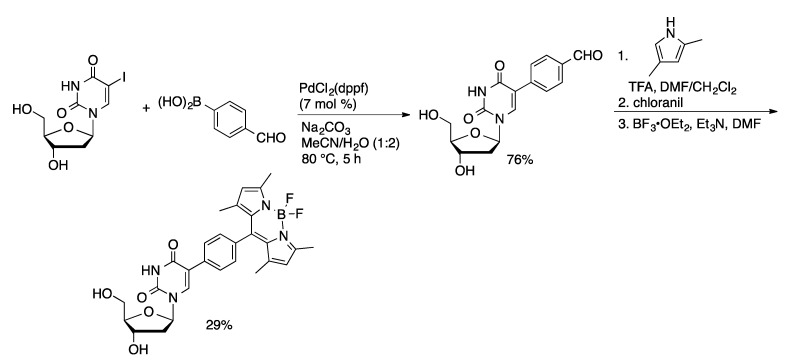
Synthesis of BODIPY-functionalized uridine.

1,1′-Bis(diphenylphosphino)ferrocene (dppf) has also been applied as a ligand in the Suzuki coupling of unprotected nucleosides. Wagenknecht used PdCl_2_(dppf) (10 mol %) to catalyze the coupling of 10-methylphenothiazin-3-ylboronate ester **1** with 5-IdU in THF/MeOH/H_2_O (2:1:2) to provide the coupled product in 34% yield (Scheme 13) [51]. This method afforded 5-(2-pyrenyl)dU in 62% yield from 5-IdU and pinacol pyrene-2-boronate ester [52]. A route to BODIPY-modified uridines starts with the PdCl_2_(dppf)-catalyzed coupling of 4-formylboronic acid with 5-IdU in water/acetonitrile (2:1) to give the product in 76% yield (Scheme 14) [53]. The aldehyde was then condensed with 2,4-dimethylpyrrole, followed by complexation with BF_3_ to afford the fluorescent uridine derivative. 2-Pyrenyl-dU was prepared in 65% by the Suzuki coupling of 2-pyrenylboronate pinacol ester with 5-IdU PdCl_2_(dppf) (11 mol %) in THF/MeOH/H_2_O (2:1:1) [47].

### 3.2. Stille Coupling

**Scheme 15:**
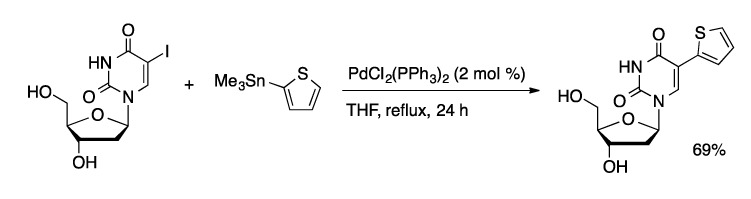
Stille coupling of 2-thienylstannane and 5-IdU.

The Stille coupling is an effective and mild method to introduce a wide range of carbon substituents. Because of the toxicity of stannanes, and the challenges associated with removing tin byproducts, the Suzuki coupling has largely supplanted the Stille coupling. The Stille coupling provides an early example of the introduction of heteroaryl, alkenyl, and allyl substituents to the 5-position of uridine (Scheme 15) [54]. Organostannanes were coupled with 5-IdU using PdCl_2_(PPh_3_)_4_ as the catalyst in refluxing THF to give the coupled products in good yields (42%–72%). Vinylstannanes are more readily available than vinylboronic acid derivatives. A recent synthesis of 8-vinyldG relied on a Pd(PPh_3_)_4_-catalyzed Stille coupling of tributylvinylstannane and 8-BrdG in NMP at 110 °C [55].

### 3.3. Sonogashira Coupling

Alkynylation of nucleosides is another important modification strategy. The alkynyl modification can be introduced with minimal effect on DNA structure. 5-Alkynyluridine can replace thymidine bases without significant structural change to the DNA conformation, for example. The Sonogashira coupling of aryl halides with alkynes is an effective method for preparing arylacetylene derivatives. This approach has been used widely with protected nucleosides in organic solvents. Because Sonogashira couplings are often performed in polar aprotic solvents, alkynylation of unprotected nucleosides using triphenylphosphine-based catalysts were some of the first examples of direct coupling of unprotected nucleosides.

**Scheme 16:**
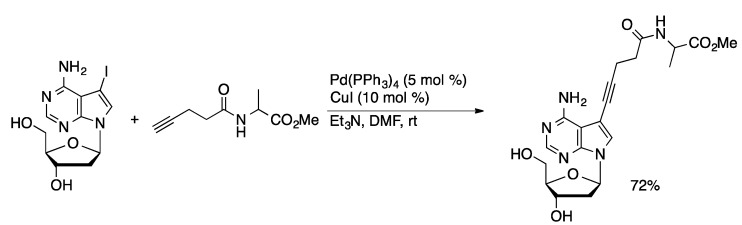
Sonogashira coupling catalyzed by Pd(PPh_3_)_4_/CuI.

The Sonogashira coupling was first demonstrated in the coupling of 2-IA with alkynes in DMF catalyzed by PdCl_2_(PPh_3_)_2_ and CuI [56]. The 2-alkynyl-A products were obtained in excellent yields (84%–97%). The versatility of the methodology has been demonstrated by preparing alkynyl-substituted nucleosides bearing acidic, basic, and hydrophobic groups from 7-I-7-deaza-dA, 8-BrdA, 7-I-7-deaza-dG, 5-IdU, and 5-IdC (Scheme 16) [57]. The functionalized nucleotides were converted to triphosphates and incorporated into oligonucleotides using polymerase enzymes. Oligonucleotides with a high density of functionalized nucleoside residues could be prepared.

**Scheme 17:**
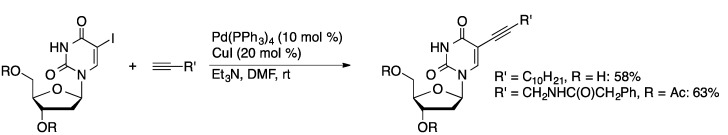
Sonogashira coupling of protected and unprotected 5-IdU.

**Scheme 18:**
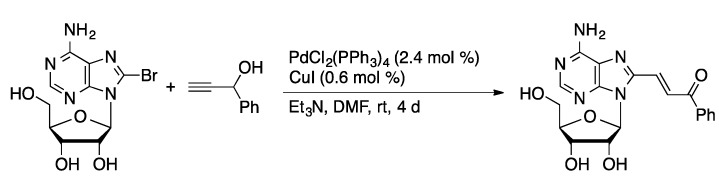
Rearrangement of 1-phenyl-2-propyn-1-ol during Sonogashira coupling.

Couplings of alkynes and 8-BrdA catalyzed by PdCl_2_(PPh_3_)_2_ and CuI gave a series of 8-alkynyldA derivatives [58]. The series was further diversified by reducing the alkynyl-dG compounds to 8-alkenyldG and 8-alkyldG derivatives. 8-EthynyldA, prepared from trimethylsilylacetylene followed by deprotection, was the most active of the compounds with micromolar inhibitory activity against a range of viral targets. Sonogashira coupling catalyzed by Pd(PPh_3_)_4_ in DMF at rt was used to prepare 5-alkynyl-dU derivatives containing lipophilic, amide, urea, and sulfonamide functionality [45]. Simple aliphatic alkynes, such as 1-dodecyne, were coupled directly with 5-IdU (Scheme 17). Alkynes with polar functionalities were prepared by coupling of acetoxy-protected 5-IdU with alkynes. The authors do not indicate why protected 5-IdU was used with the functionalized alkynes. The library of compounds was tested as inhibitors of mycobacterial thymidylate synthases (ThyX and ThyA) in *Mycobacterium tuberculosis*. 8-Alkynyl-A derivatives, prepared by the PdCl_2_(PPh_3_)_2_/CuI-catalyzed coupling of 8-BrA and alkynes, gave selective antagonists of the A_3_ adenosine receptor [59]. An attempt to prepare 8-(3-phenyl-3-hydroxypropyn-1-yl)A resulted in rearrangement of the alkyne to give 8-(3-phenyl-1-propyn-3-one)A instead (Scheme 18).

**Scheme 19:**
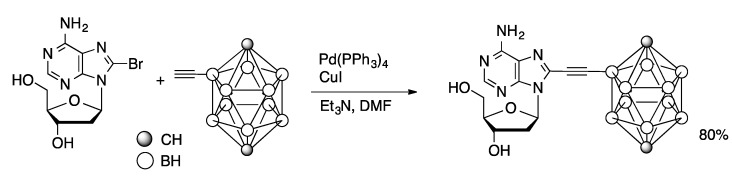
Sonogashira coupling of 8-BrdA with 2-ethynyl-*para*-carborane.

**Scheme 20:**
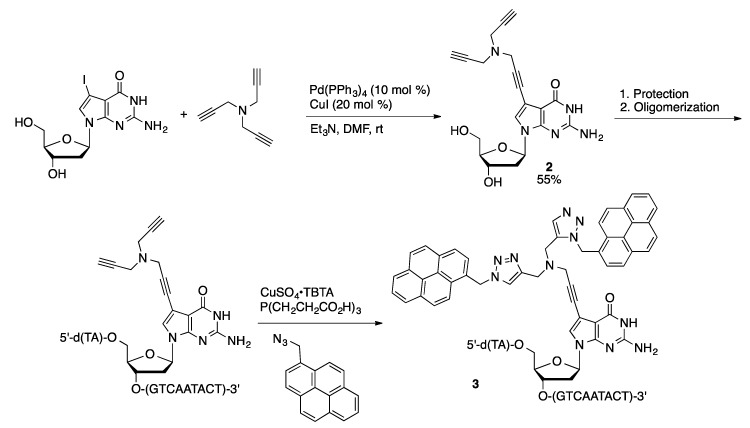
Synthesis of doubly pyrene-substituted oligonucleotides.

Boron-rich compounds are attractive pharmacophores of use in boron neutron capture therapy. Coupling of 8-BrdA with 2-ethynyl-*para*-carborane catalyzed by Pd(PPh_3_)_4_ and CuI in DMF afforded the 8-substituted adenosine in 80% yield (Scheme 19) [60]. Alternatively, 8-ethynyl-dA could be coupled with 2-iodo-*para*-carborane, but in only 27% yield. Coupling of tripropargyl amine (10 equivalents) and 7-I-7-deaza-dG catalyzed by Pd(PPh_3_)_4_ and CuI in DMF gave a 7-alkynylated product **2** with two free alkynyl units (Scheme 20) [61]. Guanosine derivative **2** was converted to a protected phosphoramidite and incorporated into oligonucleotides using solid phase synthetic techniques. The oligonucleotides were then reacted with 1-azidomethylpyrene to give the doubly functionalized structure. Single strand oligonucleotides containing residue **2** (**3**) do not show excimer fluorescence, nor do double strand (ds) DNA containing only one strand with residue **2**. In contrast, ds oligonucleotides containing two appropriately placed **2** residues show strong excimer excitation.

### 3.4. Heck Couplings

**Scheme 21:**
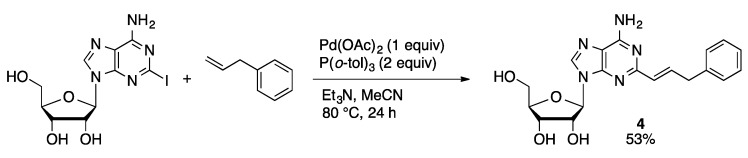
Heck coupling of 2-IA mediated by Pd(OAc)_2_/P(*o*-tolyl)_3_.

**Scheme 22:**
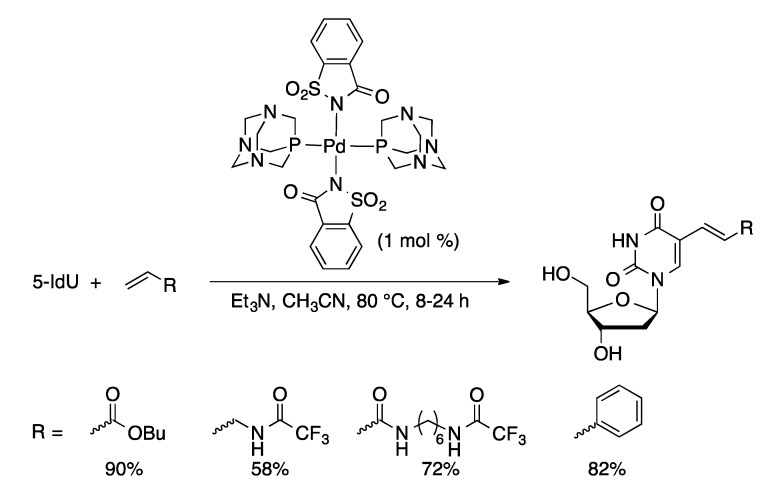
Heck coupling of 5-IdU catalyzed by Pd(PTA)_2_(saccharinate)_2_.

**Figure 2:**
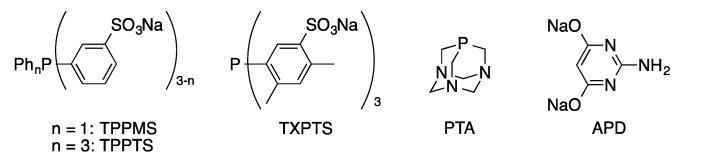
Hydrophilic ligands commonly applied in nucleoside cross-coupling reactions.

In contrast to the other classic palladium-catalyzed C–C bond-forming reactions, there are limited examples of the Heck coupling of unprotected nucleosides. Baranger reported the coupling of 2-IA with allylbenzene mediated by a stoichiometric amount of Pd(OAc)_2_/P(*o*-tolyl)_3_ in acetonitrile (Scheme 21) [62]. 2-(3-Phenyl-1-propenyl)adenosine (**4**) was isolated in 53% yield. Compound **4** was then hydrogenated to give 2-(3-phenylpropyl)A. Palladium(PTA)_2_(saccharinate)_2_ (PTA = 1,3,5-triaza-7-phosphaadamantane, Figure 2) is an effective precatalyst for the Heck coupling of 5-IdU and alkenes in acetonitrile to give 5-alkenylated dU derivatives in high yield (Scheme 22) [63]. The saccharinate complex was more active than other imidate PTA complexes (phthalimidate, maleimidate, or succinimidate).

## 4. Aqueous-Phase Cross-Coupling of Nucleosides, Nucleotides, and Oligonucleotides Using Hydrophilic Ligand-Supported Catalysts

Palladium-catalyzed cross-coupling reactions are typically performed in organic solvents using hydrophobic supporting ligands, such as triphenylphosphine. For typical hydrophobic substrates, these homogeneous conditions typically offer optimal catalyst activity. Aqueous-biphasic using water-soluble transition metal catalysts offers a number of potential advantages over traditional homogeneous organic-phase catalysis [64,65,66,67]. Water is an attractive solvent as it is non-toxic, non-flammable, and a renewable resource. Separation of homogeneous catalysts from organic products is a common challenge, particularly in pharmaceutical processes [68]. The potential to constrain the catalyst in the aqueous phase allows for easily separation from the organic products. The standard approach to design water-soluble ligands is to append hydrophilic functionality to commonly used ligand structures, such as triphenylphosphine (Figure 2). Hydrophilic catalysts have primarily been applied in coupling of hydrophobic substrates. They also provide the opportunity to perform homogeneous coupling of hydrophilic substrates, such as biomolecules.

Casalnuovo [69] was the first to report the application of a water-soluble palladium/phosphine catalyst for the cross-coupling of aryl halides. He showed that Pd(TPPMS)_3_ (TPPMS = sodium diphenyl(3-sulfonatophenyl)phosphine) provided an effective catalyst for Suzuki, Heck, and Sonogashira couplings of aryl and heteroaryl halides in aqueous acetonitrile. In addition to hydrophobic substrates, examples of Heck and Sonogashira couplings of 5-IdU and 5-IdCMP were reported. By using a water-soluble catalyst system, cross-coupling of these hydrophilic substrates was accomplished under homogeneous conditions. In the decade following Casalnuovo’s report, significant effort was devoted to developing new water-soluble phosphine ligands and their application to cross-coupling of water-insoluble substrates. In contrast, no examples of the application of water-soluble catalyst systems to nucleoside modification were reported in the decade following Casalnuovo’s seminal paper.

### 4.1. Nucleosides

#### 4.1.1. Suzuki Coupling

*Methodology Development*. The Shaughnessy group revisited the aqueous-phase cross-coupling of nucleosides with the goal of making this a general methodology for modification of both purine and pyrimidine nucleosides. Casalnuovo’s initial paper reported coupling of the more reactive iodopyrimidine nucleosides, whereas we had an interest in coupling the less reactive 8-bromopurines, such as 8-Br(d)G and 8-Br(d)A. Catalysts derived from a range of hydrophilic phosphine ligands were screened for the ability to couple phenylboronic acid and 8-BrdG in aqueous acetonitrile [70]. The sterically demanding, electron-rich phosphine *t*-Bu-Pip-phos (4-(di-*tert*-butylphosphino)-*N,N*-dimethylpiperidinium chloride), which provides high activity catalysts for simple aryl bromides [71], gave low conversion. Palladium in combination with TPPTS, provided an effective catalyst for this coupling despite being much less effective than *t*-Bu-Pip-phos in the aqueous-phase coupling of simple aryl halides.

**Scheme 23:**
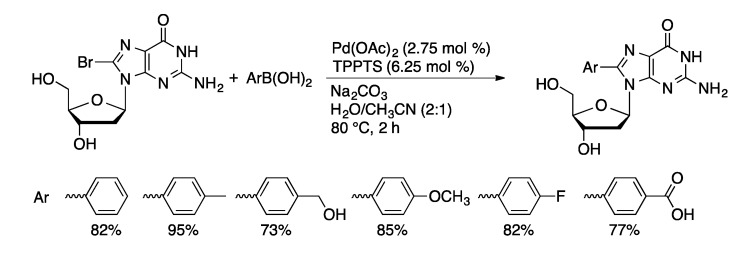
Pd(OAc)_2_/TPPTS-catalyzed Suzuki coupling of 8-BrdG.

The Pd(OAc)_2_/TPPTS catalyst system is a general method for Suzuki coupling of halogenated purine and pyrimidine nucleosides and 2′-deoxynucleosides. Good to excellent yields are afforded with a range of aryl boronic acids and 5-IdU, 8-BrG, 8-BrdG, 8-BrA, and 8-BrdA (Scheme 23) [70]. The methodology has also been extended to heteroarylboronic acids [72]. The order of reactivity of halonucleosides is 5-IdU > 8-BrdA >> 8-BrdG. The low reactivity of guanosine is believed to be due to competitive coordination of the deprotonated form of guanosine to palladium through the anionic N-1 position [73].

The more sterically demanding TXPTS ligand provides a more active catalyst for Suzuki coupling of halonucleosides [70]. Using 10 mol % Pd/TXPTS, complete conversion of 8-BrdA to 8-PhdA was achieved at room temperature in 30 minutes. In comparison, the TPPTS-derived catalyst required 24 h to give 74% conversion to product. Even 8-BrdG gave 40% conversion to 8-PhdG after 18 h at room temperature. Although TXPTS provides a more active catalyst, the Pd(OAc)_2_/TPPTS catalyst has become the standard system for these reactions. TPPTS is more widely commercially available and costs about half of TXPTS on a per mole basis [74]. For challenging cases, TXPTS may prove to be an attractive alternative.

Organic cosolvents are not required in these reactions. Good yields can be achieved using water as the only solvent [70,75,76]. In the case of the more reactive 5-IdU, the TPPTS ligand is not required [77]. Good yields could be achieved with electron-rich arylboronic acids, but much lower yields were obtained with electron-deficient boronic acids. Using TPPTS, good yields can be achieved with a broad range of arylboronic acids in water alone.

**Scheme 24:**
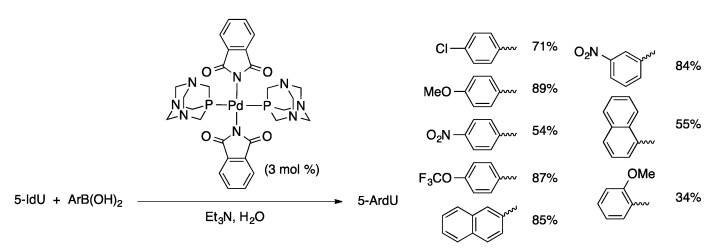
Pd(PTA)_2_(phthalimidate)_2_-catalyzed Suzuki coupling of 5-IdU.

**Scheme 25:**
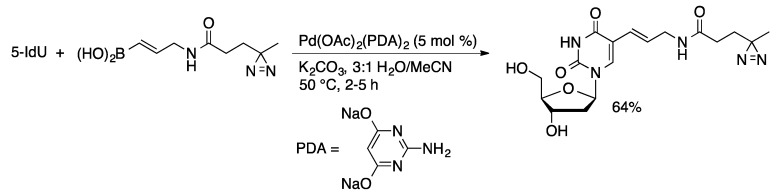
Suzuki coupling of 5-IdU catalyzed by Pd-APD complexes.

Other hydrophilic ligands have been used in the Suzuki coupling of halonucleosides with boronic acids in aqueous media. Pd(PTA)_2_(phthalimidate)_2_ (PTA = 1,3,5-triaza-7-phosphaadamantane, Figure 2) is an effective precatalyst for the synthesis of 5-aryl-2′-deoxypyridimine derivatives from 5-IdU and 5-IdC in water (Scheme 24) [78]. The phthalimidate complex gave higher yields than dihalide palladium-TPA complexes or the catalyst generated *in situ* from Pd(OAc)_2_ and PTA. Suzuki coupling of aryl- and alkenylboronic acids with 5-IdU can also be accomplished using Pd(OAc)_2_(2-aminopyrimidine-4,6-diolate)_2_ as the precatalyst (Scheme 25) [79]. These catalyst systems have not been extended to the less reactive 8-halopurine nucleosides.

*Applications*. The palladium/TPPTS methodology sparked interest in the direct functionalization of unprotected nucleosides in aqueous reaction media. The homogeneous conditions have proven to be more general than catalyst systems using hydrophobic ligands in water or polar organic solvents. This generality is particularly useful in the coupling of nucleotide and oligonucleotide substrates as discussed in Section 4.2 and Section 4.3. The Pd/TPPTS catalyst system has been applied to the synthesis of wide variety of modified nucleosides that incorporated fluorescent or electrochemically active reporter groups, coordination sites, or have potential pharmaceutical activity.

**Scheme 26:**
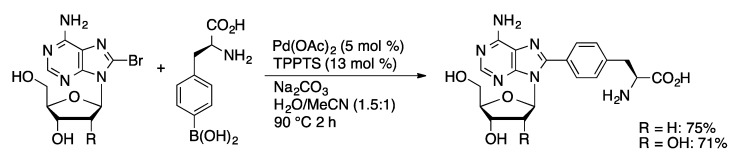
Pd/TPPTS-catalyzed coupling of 8-bromoadenosines with phenylalanine.

**Scheme 27:**
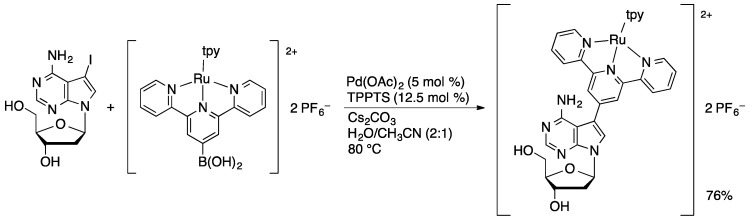
Attachment of a ruthenium(II) terpyridine complex to 7-I-7-deaza-dA.

An early demonstration of the utility of the Pd/TPPTS catalyst system was the synthesis of an amino acid-nucleoside adduct reported by Hocek [80]. The coupling of 8-BrA and 8-BrdA with phenylalanyl-4-boronic acid is achieved in 71 and 75% yield, respectively, in water-acetonitrile using the Pd/TPPTS catalyst system (Scheme 26). Notably, neither the nucleoside nor amino acid substrates contained protecting groups. In the coupling with 6-chloropurine nucleosides, improved yields were obtained using microwave irradiation compared to traditional thermal heating [81]. The Hocek group has also reported the attachment of polypyridyl ligands to 8-BrdA [82], 7-I-7-deaza-dA [83], and 5-IdU [84] via Suzuki couplings using Pd/TPPTS (Scheme 27). High yields were achieved using bipyridyl, phenanthryl, and terpyridyl boronic acids as either free ligands or preformed ruthenium complexes. The ruthenium complex-modified nucleosides are of interest as luminescent and electroactive probes in oligonucleotides.

The Pd/TPPTS catalyzed Suzuki coupling has been used to introduce a range of fluorescent probes into nucleosides. This methodology has been used to attach five-membered ring heterocycles (2-pyrrolyl, 2-indolyl, 2-furyl, and 2-thiophenyl) to the 8 position of 2′-deoxyguanosine [85,86]. The fluorescence of the resulting heterocyclic adducts is sensitive to hydrogen bonding with other nucleobases as well as the nucleoside conformation. The 8-(2-benzo[*b*]thienyl)-dG group serves as a fluorescent reporter to probe for the preference for *syn*- or *anti-*conformations in duplex DNA [87].

**Scheme 28:**
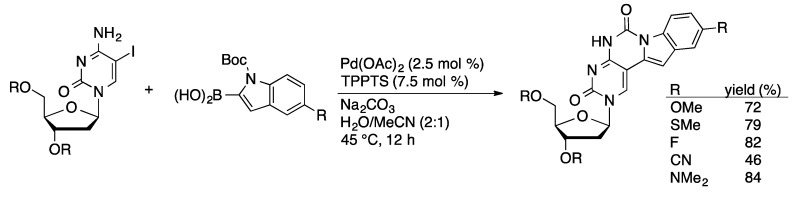
Suzuki coupling/condensation sequence to prepare pyrimidopyrimidoindole nucleosides.

**Scheme 29:**
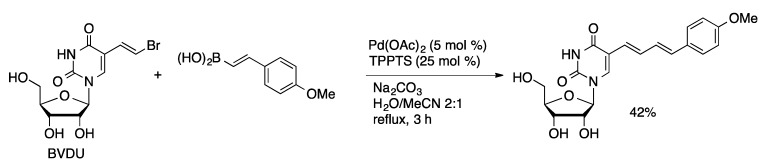
Suzuki coupling of BVDU catalyzed by Pd(OAc)_2_/TPPTS.

Fluorescent pyrrolopyrimidopyrimidine [88] and pyrimidopyrimidoindole [89] nucleosides can be prepared by aqueous-phase coupling of 5-IdC with *N*-Boc-protected 2-pyrrolylboronic acid or *N*-Boc-protected 2-indolylboronic acid (Scheme 28). The coupling is followed by condensation of the 6-amino group of the cytidine ring with the Boc moiety to give the fluorescent tri- or tetracyclic nucleoside analogs. Fluorescent 5-substituted uridine and 2-deoxyuridine analogs can be prepared by Suzuki coupling of 5-I(d)U with aryl or styrylboronic acid derivatives [90]. An alternative approach to 5-styryluridine derivatives was achieved through the coupling of arylboronic acids with commercially available 5-(2-bromovinyl)uridine (BVDU, Scheme 29).

**Scheme 30:**
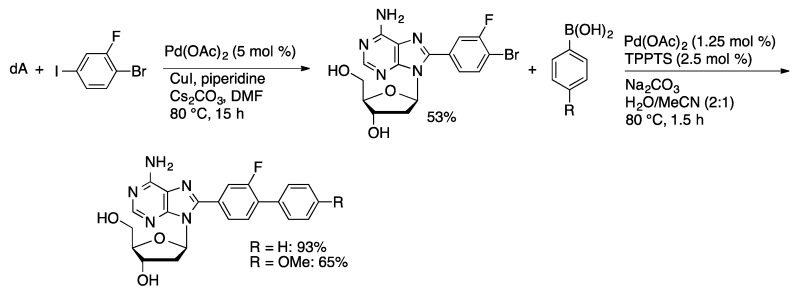
Double arylation sequence to prepare 8-biaryl-dA derivatives.

**Scheme 31:**
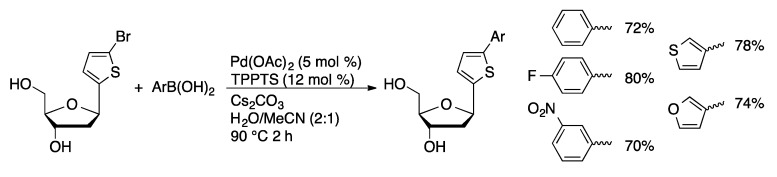
Suzuki coupling of 2′-deoxy-2′-(5-bromo-2-thiophenyl)ribose.

A library of *C*8-biaryl-modified dA derivatives were prepared by a sequence involving palladium-catalyzed oxidative coupling of iodobromobenzenes with dA (Scheme 30) [91]. The resulting 8-(bromoaryl)dA derivatives were then coupled with arylboronic acids using Pd(OAc)_2_/TPPTS (1.25 mol % Pd) in aqueous acetonitrile. Suzuki coupling of the unnatural *C*-nucleoside 2′-deoxy-2′-(5-bromo-2-thiophenyl)ribose with arylboronic acids catalyzed by Pd(OAc)_2_/TPPTS gave a series of 2-arylthiophenylribose nucleoside analogs (Scheme 31) [92].

**Scheme 32:**
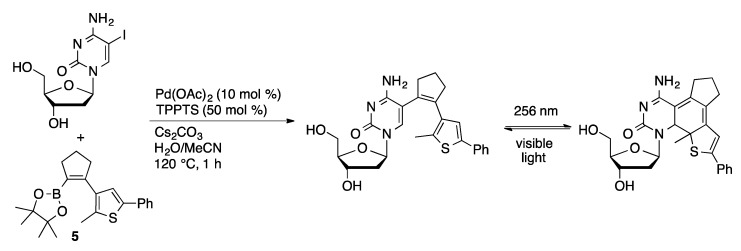
Synthesis of a photoswitchable cytidine derivative.

Suzuki coupling with Pd/TPPTS was used to introduce a photoresponsive chemical switch based on a diarylethylene moiety in pyrimidine nucleosides. Suzuki coupling of boronate ester **5** with 5-IdU and 5-IdC afforded photoswitchable nucleosides that undergo reversible photochemical electrocyclic cyclization under UV irradiation (Scheme 32) [93]. The modified nucleoside reverts to the open form under visible light. The modified nucleosides could potentially be used to photochemically control the structure and function of oligonucleotides.

**Scheme 33:**
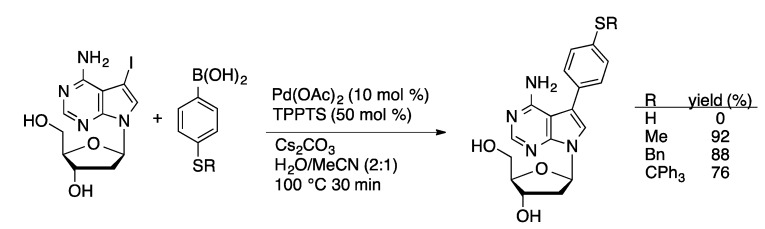
Synthesis of phenyl sulfide-substituted 7-deaza-dA derivatives.

Nucleosides containing arylthiol moieties were prepared by Suzuki coupling of protected 4-thiophenylboronic acid derivatives with 5-IdC and 7-I-7-deaza-dA using Pd(OAc)_2_/TPPTS in aqueous acetonitrile (Scheme 33) [94]. No conversion occurred in the presence of the free thiol group, but high yields were achieved with boronic acids containing protected thiols. The thiol-functionalized nucleosides can be incorporated into oligonucleotides and used to attach the DNA to gold surfaces.

Guanosine forms self-assembled tetrameric structures (G-tetrads) through intermolecular hydrogen bonding. In the presence of cations, these can further self assemble into G-quadruplexes. Structural modifications can be used to enhance this self assembly. Aqueous-phase Suzuki coupling was used to prepare 8-(3- or 4-acetylphenyl)dG derivatives [95]. The acetylphenyl moiety enhances G-tetrad formation by providing additional hydrogen bonding opportunities, while also extending the aromatic surface to improve noncovalent interactions.

**Scheme 34:**
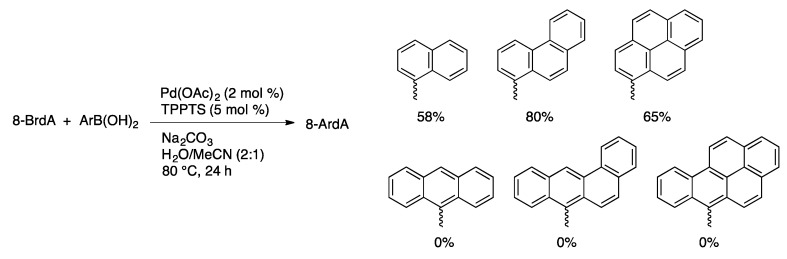
Coupling of 8-BrdA with polyaromatic boronic acids.

8-Aryl-substituted derivatives of purine nucleosides are of interest as models of adducts formed *in vivo* during the metabolism of aromatic hydrocarbons. The motivation of our original study of the aqueous-phase Suzuki coupling of 8-BrdG was to prepare adducts to study the effect of these modifications on DNA conformation [96]. The carcinogenesis of polyaromatic hydrocarbon is thought to involve covalent modification of guanosine during metabolism of these compounds *in vivo*. Suzuki coupling of 1-pyrenylboronic acid, 1-naphthylboronic acid, and 9-phenanthrenylboronic acid with 8-BrdA gave good yields of the adducts using Pd(OAc)_2_/TPPTS (Scheme 34) [5]. The more hindered 8-anthracenylboronic acid and its benzannulated analogs could not be coupled under these conditions, however.

**Scheme 35:**
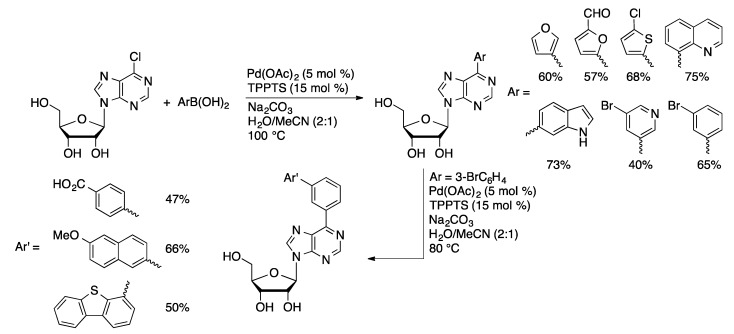
Suzuki coupling of 6-chloropurine nucleoside catalyzed by Pd(OAc)_2_/TPPTS.

Arylated nucleoside derivatives have been explored as potential pharmaceutically active compounds with antiviral and anticancer activity. The Hocek group has used the Pd(OAc)_2_/TPPTS catalyst system to prepare a wide variety of natural and non-natural nucleoside derivatives through aqueous-phase Suzuki couplings. A library of 6-heteroarylpurine nucleosides were prepared from unprotected 6-cloropurine and 6-chloro-7-deazapurine nucleosides [97,98]. Good yields (40%–80%) were achieved with a variety of aryl and heteroaryl boronic acids. A similar library was prepared from 7-I-7-deaza-A [99]. A large library of 6-arylpurine nucleoside monophosphates were prepared by aqueous-phase Suzuki coupling of 6-chloropurine nucleoside with arylboronic acids followed by phosphorylation (Scheme 35) [100]. Further elaboration of 6-(3-bromophenyl)purine nucleoside with a subsequent Suzuki coupling afforded 6-biarylpurine nucleosides. The compounds were tested for their ability to inhibit 2′-deoxynucleoside 5′-phosphate *N*-hydrolase 1 (DNPH1), which is a potential anticancer target.

**Figure 3:**
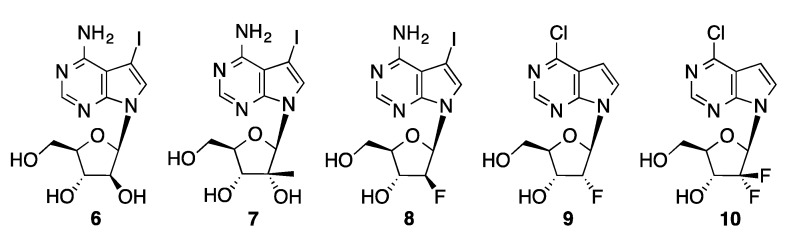
Sugar-modified purine halonucleosides.

**Scheme 36:**
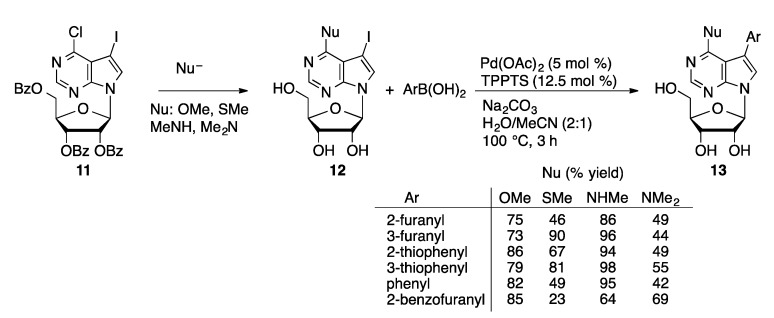
S_N_Ar/Suzuki sequence to 6-substituted-7-aryl-7-deazapurine nucleosides.

The Hocek group has also used aqueous-phase Suzuki couplings to prepare arylated nucleoside derivatives in which the ribose unit has been modified. Good yields are obtained in coupling of aryl and heteroarylboronic acids with 7-iodo-7-deazaadenine arabinoside (**6**) [101], 7-iodo-7-deaza-2′-*C*-methyladenosine (**7**) [101], 7-iodo-7-deazaadenine 2′-deoxy-2′-fluoroarabinoside (**8**) [101], 6-chloro-7-deazapurine 2′-deoxy-2′-fluororibinoside (**9**) [102], and 7-iodo-7-deazaadenine 2′-deoxy-2′,2′-difluoro-β-d-*erythro-*pentofuranoside (**10**, Figure 3) [102]. The sugar modifications have little effect on the Suzuki coupling reaction. A family of 6-substituted-7-aryl-7-deazapurinenucleosides (**13**) was prepared starting from a protected 6-chloro-7-iodo-7-deazapurine nucleoside derivative (**11**, Scheme 36) [103]. Selective nucleophilic aromatic substitution at the 6-chloro position followed by deprotection provides the 7-iodo precursors (**12**). These were coupled with aryl or heteroarylboronic acids using Pd(OAc)_2_/TPPTS.

#### 4.1.2. Sonogashira Coupling

**Scheme 37:**
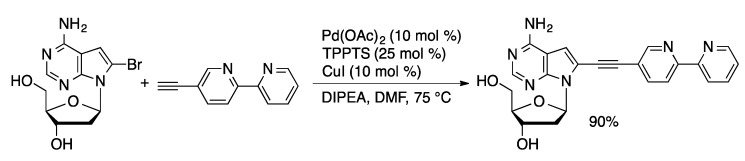
Sonogashira coupling approach to bipyridine-dA adduct.

Palladium-catalyzed alkynylation of unprotected nucleosides with hydrophobic palladium/phosphine catalysts is well precedented. Recently, aqueous-phase Sonogashira couplings using hydrophilic ligands has received increasing attention. The Hocek group used Sonogashira couplings, in addition to Suzuki couplings, to introduce polypyridyl ligands to 8-BrdA (Scheme 37) [82], 7-I-7-deaza-dA [83], and 5-IdU [84] using the Pd/TPPTS catalyst system in combination with catalytic CuI in DMF. High yields were achieved with bipyridyl alkynes (82%–96%). In contrast to the Suzuki coupling, low yields were obtained in the coupling of ruthenium-coordinated analogs of the bipyridinyl alkynes (0%–57%). Decomposition of the alkyne-substituted ruthenium complexes competed with the desired cross-coupling under the Sonogashira reaction conditions.

**Scheme 38:**
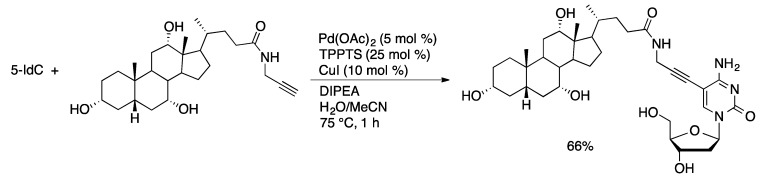
Synthesis of bile acid-dC adducts through aqueous-phase Sonogashira coupling.

Sonogashira coupling with Pd(OAc)_2_/TPPTS/CuI is effective for the coupling of 7-I-7-deaza-dA and 5-IdU with propargyl esters or amides of bile acids (Scheme 38) [104]. Yields of isolated products ranged from 31%–90%. The bile acid nucleoside adducts are of interest for oligonucleotide amphiphiles.

**Scheme 39:**
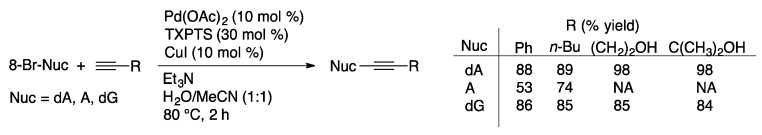
Pd/TXPTS-catalyzed Sonogashira coupling of 8-bromopurine nucleosides.

**Scheme 40:**
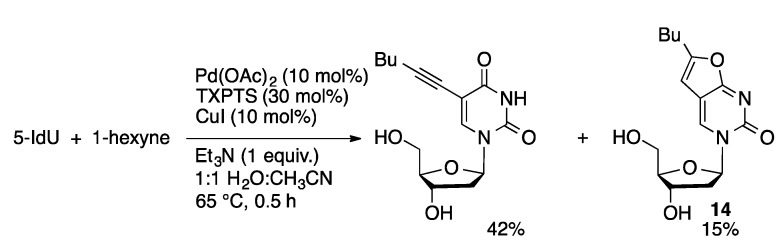
Pd/TXPTS-catalyzed Sonogashira coupling of 5-IdU.

The more sterically demanding TXPTS ligand provides a more active catalyst for the Sonogashira coupling of 8-halopurines and 5-IdU than the catalyst derived from TPPTS [105,106]. In the coupling of 5-IdU with phenylacetylene catalyzed by TPPTS, Pd(OAc)_2_ and CuI in aqueous acetonitrile reached 80% conversion after six hours at 50 °C. Under the same conditions, 90% conversion was achieved in one hour using TXPTS as the ligand. The Pd(OAc)_2_/TXPTS system was effective for the coupling of aryl and alkyl acetylenes with 8-Br(d)A and 8-BrdG (Scheme 39). In the case of 5-IdU, the Sonogashira reaction was followed by nucleophilic attack of the C6-O on the alkyne resulting in formation of furano [2,3,]-pyrimidin-2-one byproducts (**14**, Scheme 40).

#### 4.1.3. Heck Couplings

There are relatively few examples of Heck couplings of unprotected nucleosides reported with hydrophilic ligands. The Shaughnessy group reported the Heck coupling of 5-IdU with styrene and conjugated enones [107]. The Pd(OAc)_2_/TPPTS gave good yields of 5-alkeynyl products, but similar yields were obtained when no ligand was used. More sterically demanding (TXPTS) or electron-rich ligands (*t*-Bu-Amphos) did not improve the catalyst performance. The Pd(OAc)_2_/TPPTS catalyst was successfully used in the synthesis of 8-styryl-dG starting from 8-BrdG at 80 °C in 2:1 water/acetonitrile [55].

### 4.2. Nucleotides

Modified nucleosides are often prepared as precursors to nucleotides, which can be enzymatically incorporated into oligonucleotides. Selective 5′-phosphorylation of modified nucleosides represents one common approach to preparing base-modified nucleotides. Phosphorylation of lipophilic modified nucleosides can be difficult to achieve in high yield and good selectivity for the 5′-oxygen [108]. An alternative approach is to perform the cross-coupling reaction directly on the halogenated nucleotide. Water-soluble catalyst systems have proven to be effective in performing cross-coupling reactions with nucleoside mono-, di-, and triphosphate substrates. In contrast, there are no reported examples of the use of catalysts derived from hydrophobic ligands in the direct reaction of halogenated nucleotides.

#### 4.2.1. Suzuki Coupling

**Scheme 41:**
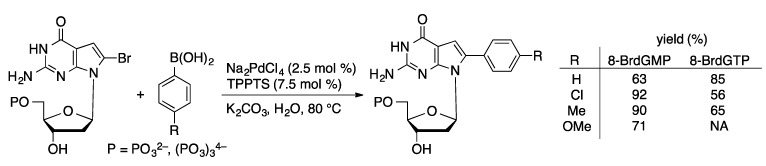
Pd/TPPTS-catalyzed Suzuki coupling of 8-bromoguanosine phosphates.

The Pd/TPPTS catalyst system that is widely used for nucleoside Suzuki couplings is also effective in couplings with halogenated nucleotide derivatives. Early examples of Suzuki couplings of halonucleotides were reported by the Wagner [109] and Hocek groups [81,110]. Both groups used a catalyst derived from a palladium(II) salt and TPPTS (Scheme 41). Notably, the reaction could be carried out at a high pH (0.3 M CO_3_^2−^) at an elevated temperature (80–120 °C) without decomposition of the nucleotide.

**Scheme 42:**
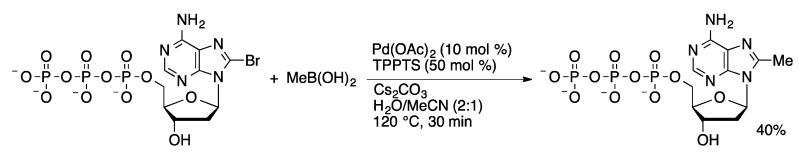
Methylation of 8-BrATP.

Hocek reported similar conditions for the synthesis of 8-MedATP and 8-PhdATP from 8-BrdATP [111]. The methyl-substituted nucleoside was prepared using methylboronic acid, which is a rare example of the use of an alkylboron reagent in coupling reactions with nucleoside derivatives (Scheme 42). The resulting modified nucleosides could be incorporated into oligonucleotides using polymerase enzymes. This methodology was used to prepared 5-ArdUTP, 5-ArdCTP, and 7-Ar-7-deazadATP derivatives with 3-nitrophenyl and 3-aminophenyl aryl groups [112]. The modified nucleotides were incorporated into oligonucleotide sequences. The modifications serve as electrochemical labels in oligonucleotides that are sensitive to the local sequence.

These conditions have been applied to the synthesis of a variety of functionalized nucleotides that were then incorporated into oligonucleotides using polymerase enzymes. Alkylsulfanylphenyl-modified nucleotides were prepared modes yields (10%–50%) by coupling of the thioether-functionalized boronic acids with 7-I-7-deaza-dATP and 5-ICTP using the Pd/TPPTS catalyst system [94]. The resulting functionalized nucleotides were enzymatically incorporated into oligonucleotides. The functionalized oligonucleotides were studied electrochemically and found to associate at the gold electrode surface.

**Scheme 43:**
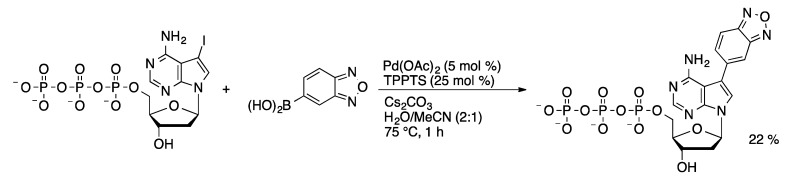
Synthesis of benzofurane-dATP.

Benzofurane has been proposed as a novel electrochemically active probe functionality for the study of oligonucleotides. The benzofurane moiety was attached to 7-deaza-dATP and dCTP via an aqueous-phase Suzuki coupling catalyzed by Pd/TPPTS [113]. Low yields (10%–22%) were obtained for the Suzuki coupling of the halonucleoside triphosphate substrates (Scheme 43). Suzuki coupling of the halonucleoside followed by phosphorylation gave a higher overall yield (17%–52%) of the benzofurane nucleotide adducts. Electrochemically active benzofurane moieties were attached to nucleotides in incorporated into oligonucleotides in parallel with nitrophenyl- and aminophenyl-modified nucleotides. The three electroactive groups could be addressed independently without apparent interference.

**Scheme 44:**
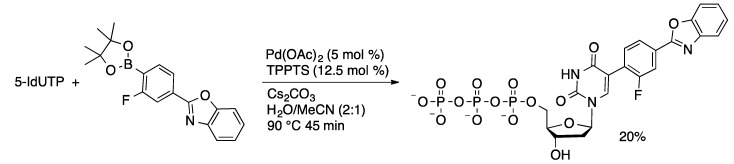
Synthesis of pH-sensitive dual fluorescent ^19^F-NMR probe nucleotides.

**Scheme 45:**
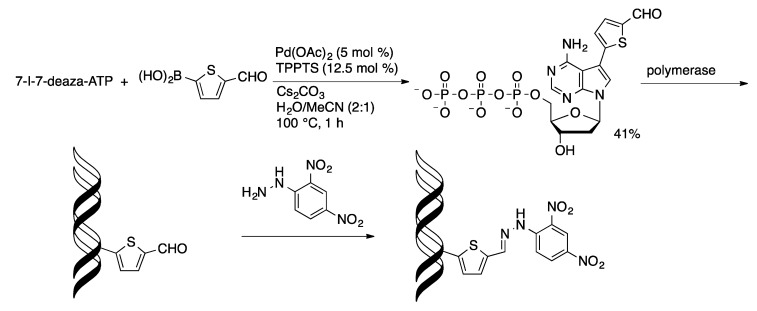
Synthesis of aldehyde-functionalized nucleoside triphosphates.

Solvatochromatic and pH-sensitive dual fluorescent ^19^F-NMR probes were attached to 7-I-7-deaza-dATP and 5-IdUTP using an aqueous-phase Suzuki coupling (Scheme 44) [114]. The nucleotides were incorporated into oligonucleotides where they showed environment-dependent fluorescence properties. The Pd/TPPTS catalyst was used to prepare 5-formylthiophen-2-yl-modified nucleotides, which were then incorporated into oligonucleotides via primer extension or polymerase chain reaction protocols (Scheme 45) [115]. The aldehyde-modified oligonucleotides were then conjugated with amine derivatives through the formation of imine linkages. This methodology was used for selective staining of the aldehyde-containing oligonucleotides.

**Scheme 46:**
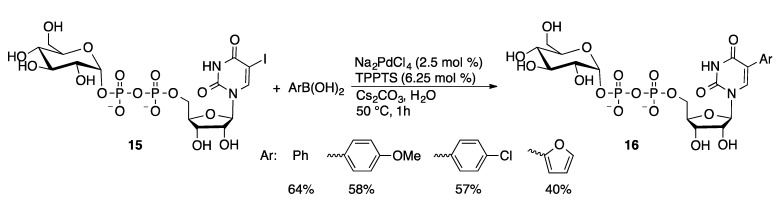
Arylation of 5-I-UDP glucoside.

**Scheme 47:**
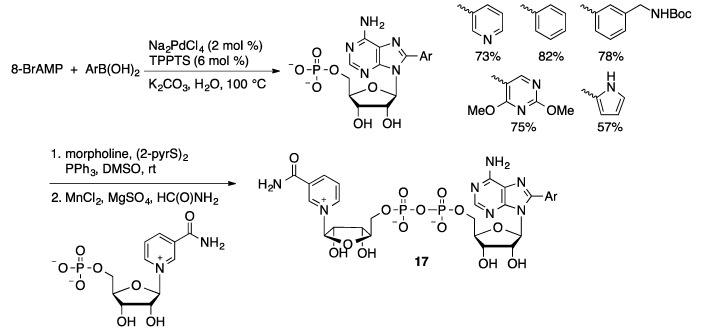
Synthesis of 8-arylated nicotinamide adenine dinucleotides.

The aqueous-phase Suzuki coupling protocol has also been applied to the synthesis of dinucleotides and nucleotide-sugar conjugates. A series of arylboronic acids were coupled with 5-I-UDP glucoside (**15**) in water catalyzed by Na_2_PdCl_4_/TPPTS (1 mol %) to give 5-arylated products **16** in 40%–64% yield (Scheme 46) [116]. The corresponding 5-Br-UDP-glucose substrate gave no conversion under identical conditions. Analysis of the nucleotide conformation by NOE spectroscopy showed that 5-Br-UDP-glucose preferred an *anti*-configuration that places the bromide over the ribose ring, potentially making it less accessible. In contrast, 5-I-UDP-glucose preferred a *syn*-conformation in which the iodide is more readily accessible to the catalyst. It should also be noted that C–I bonds are generally found to be more reactive in cross-coupling reactions than C–Br bonds. A similar methodology was used to prepare 8-aryl-GDP-mannoside derivatives in 48%–82% yield for the Suzuki coupling step [117]. 8-Arylated nicotinamide adenine dinucleotides (**17**) were prepared by an alternate approach in which 8-BrAMP was arylated using Pd/TPPTS (Scheme 47) [118]. The arylated AMP was then coupled to nicotinamide ribose monophosphate through a phosphorylation reaction. The resulting compounds serve as fluorescent probes for NAD-consuming enzymes.

#### 4.2.2. Sonogashira Coupling

**Scheme 48:**
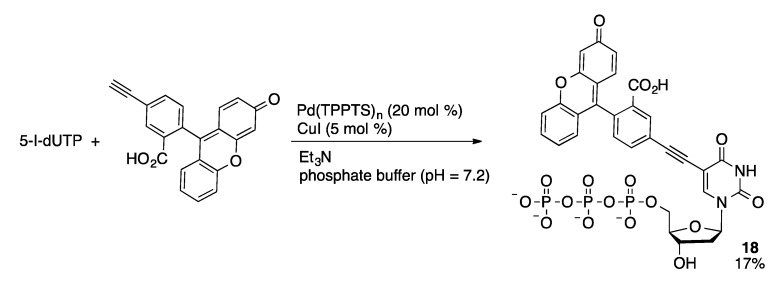
Sonogashira coupling of 5-I-dUTP and ethynylfluorescein.

Sonogashira coupling of alkynes with halogenated nucleotides is an effective method to introduce probe species. Burgess was the first to demonstrate this methodology in the synthesis of fluorescent dye-modified uridine triphosphates (**18**, Scheme 48) [108]. Fluorescein-based dyes with alkyne substituents were coupled with 5-I-dUTP using a preformed Pd/TPPTS catalyst and CuI at room temperature with triethylamine as the base in phosphate buffer. The preformed catalyst was generated by mixing Na_2_PdCl_4_, TPPTS, NaBH_4_ and water at room temperature followed by removal of water and recovery of the resulting solid. Alkynylation of the nucleotide substrate was necessary to produce the desired compounds. In contrast, attempted phosphorylation of fluorescein-modified dU was unsuccessful.

**Scheme 49:**
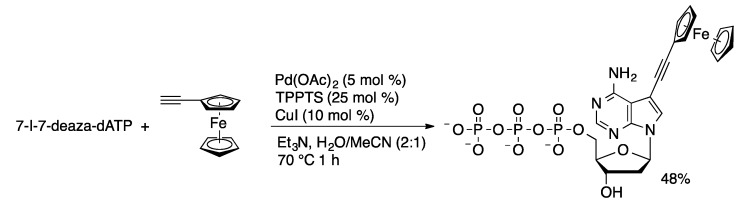
Synthesis of ferrocenyl-modified 7-deaza-dATP.

Phenylalanyl-modified nucleotide triphosphates can be prepared in 60%–67% yield by the Sonogashira coupling of 4-ethynylphenylalanine with halonucleotides using a catalyst derived from Pd(OAc)_2_, TPPTS, and CuI in aqueous acetonitrile. This method was also used to prepare ethynylferrocene-modified nucleotide triphosphates (Scheme 49) [119]. Oligonucleotides prepared from the ferrocene-functionalized nucleotides are electrochemical probes of DNA binding. Bile acid conjugates of nucleotides were prepared in moderate yields (32%–57%) by coupling of propargyl bile acid amides with iodonucleotide triphosphates [104].

#### 4.2.3. Heck Coupling

**Scheme 50:**
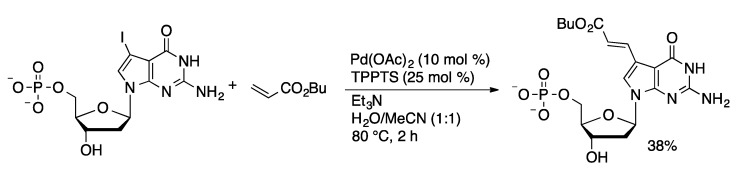
Aqueous-phase Heck coupling of 7-iodo-7-deazaguanosine monophosphate.

To date there is only one example of a Heck coupling of a halonucleotide. Hocek reported the coupling of butyl acrylate with a range of 5-iodopurine and 7-iodo-7-deazapurine nucleoside mono- and triphosphates using Pd(OAc)_2_ and TPPTS in water/acetonitrile at 80 °C (Scheme 50) [120]. The yields with nucleotides were modest (14%–55%) compared to the high yields obtained with the corresponding nucleosides (81%–98%). The monophosphates gave higher yields than triphosphates. Although modest yields were obtained in the Heck coupling of nucleotides, the yields were similar to those obtained when the Heck coupling was carried out on the nucleoside followed by phosphorylation. Cytidine gave low yields in the Heck coupling and no conversion was obtained with cytidine mono- or triphosphate. The resulting alkene-modified nucleotides could be successfully incorporated into oligonucleotides by primer extension methods.

### 4.3. Oligonucleotides

Palladium-catalyzed coupling reactions provide effective ways to attach a wide variety of moieties to nucleoside or nucleotide structures. The modified nucleosides can in many cases be effectively incorporated into oligonucleotides using primer extension, PCR, or solid-phase DNA synthesis methods. Although these approaches to modified oligonucleotides have been demonstrated, significant limitations to these approaches have been observed. For example, C8-arylated purine nucleosides are significantly more prone to acidic hydrolysis of the glycosidic bond and are more sensitive to oxidation than dA or dG [121]. As a result, they often are not compatible with solid-phase DNA synthesis techniques. A variety of modified nucleotide derivatives have been incorporated into oligonucleotides through enzymatic polymerase approaches, but steric limitations can limit the effectiveness of this approach [110,111].

A more general approach to preparing oligonucleotides with modified bases would be to build the nucleotide containing halogenated residues at the desired locations, followed by post-synthetic palladium-catalyzed coupling of the halonucleosides. The successful coupling of nucleotides suggests that the oligonucleotide backbone should be stable in the coupling reaction. An oligonucleotide represents a significantly more complex substrate than a simple nucleotide monomer, however. Successful development of this approach would allow a variety of modified oligonucleotides to be prepared from a common precursor containing a halogenated base residue at the desired position on the oligonucleotide.

**Scheme 51:**
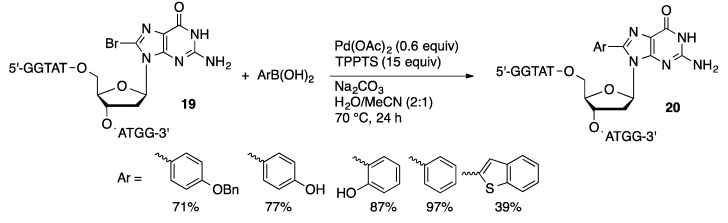
Suzuki coupling of 8-BrdG-containing oligonucleotide.

Manderville reported the first successful example of the post-synthetic cross-coupling of a halogenated oligonucleotide [121]. Solution-phase coupling of oligonucleotides containing a single 8-bromoguanosine residue with arylboronic acids was performed with Pd(OAc)_2_/TPPTS in water/acetonitrile with Na_2_CO_3_ as base (Scheme 51). Under optimized conditions, a guanine rich decanucleotide containing 8-BrdG (**19**) was arylated with 2-hydroxyphenylboronic acid to give **20** in 87% yield. In contrast, an attempt to prepare the same 8-arylguanine-containing decanucleotide by traditional solid-phase DNA synthetic methods resulted in the formation of a mixture of products. The major products were truncated oligomers that did not incorporate the 8-arylguanosine residue. Subsequent studies showed that a maximum of two bases could be incorporated after the 8-arylguanosine residue in the oligomerization process. The post-synthetic coupling approach was applied to the synthesis of oligonucleotides with up to 15 bases containing a single 8-BrdG residue.

**Scheme 52:**
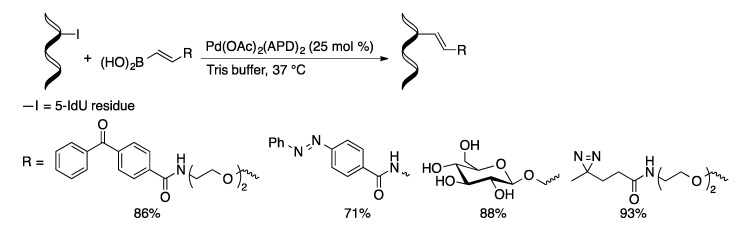
Suzuki vinylation of 5-IdU-containing oligonucleotides.

The post-synthetic approach was applied to the synthesis of oligonucleotides containing diarylethylene photoswitches [93]. Oligonucleotides (15- and 19-mers) containing 5-IdC or 5-IdU residues were coupled with boronic acid **5** with Pd(OAc)_2_/TPPTS in aqueous acetonitrile at 120 °C to give the coupled oligonucleotides in modest yields (16%–35%). The hindered boronic acid (**5**) is a much more challenging substrate than those used by Manderville. Coupling of oligonucleotides containing 5-IdU with vinylboronic acids occurs in high yields (49%–95%) using a preformed palladium complex of 2-aminopyrimidine-4,6-diolate (APD) as the precatalyst (Scheme 52) [79]. With the less-hindered boronic acids, the coupling could be performed under mild conditions (23–37 °C) in phosphate buffer. The reaction was highly selective with only small amounts of deiodination observed in some cases.

**Scheme 53:**
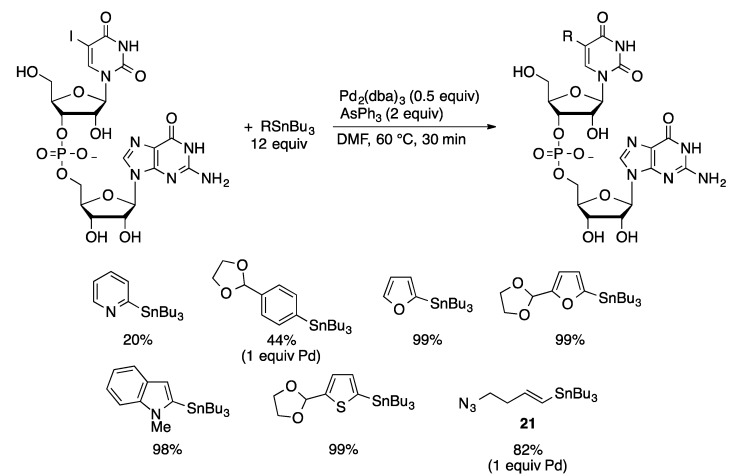
Stille coupling of 5-I-UG.

The increased hydrolytic sensitivity of RNA makes it an even more challenging substrate than DNA for cross-coupling reactions, which often require strongly basic conditions. The Stille coupling can be carried out under mild conditions. Stille coupling of the dinucleotide 5-IUpG was catalyzed by Pd_2_(dba)_3_/AsPh_3_ (50 mol % Pd) in DMF at 60 °C (Scheme 53) [122]. Excellent yields were obtained with electron-rich arylstannanes. This methodology was applied to the arylation of RNA oligomers containing a 5-IU residue supported on CPG solid support. Solid-supported 5-I-UAUAGGAGCU with stannane **20** gave the coupled product in 59% yield after removal from the solid support with ammonia.

Although only a few examples have been reported to date, the post-functionalization of oligonucleotides offers an exciting opportunity to prepare modified RNA and DNA structures. To date, only the Suzuki and Stille coupling of halogenated oligonucleotides has been demonstrated. Extension of this methodology to other important coupling reactions, such as the Sonogashira coupling, will further expand the usefulness of this transformation.

## 5. Conclusions

Palladium-catalyzed cross-coupling has developed into a highly effective method for the modification of unprotected nucleosides through carbon–carbon bond-forming reactions. Through the use of polar media, such as water, polar aprotic solvents, or combinations of the two, the hydrophilic nucleosides can be solubilized and converted to the desired adducts without the need to convert the nucleoside to a more lipophilic form. The ability to directly functionalize the nucleoside increases the overall yield and atom efficiency of the synthesis by avoiding the protection/deprotection sequence. In addition, protection strategies are generally not possible with the more hydrophilic nucleotide and oligonucleotide substrates. Catalysts supported with hydrophilic ligands provide the most general catalysts for modification of nucleoside derivatives. In aqueous solvents, hydrophilic catalysts are effective with nucleoside, nucleotides (mono- and triphosphates), and oligonucleotides. The ability to directly couple oligonucleotides is particularly noteworthy. Firstly, the ability to couple highly complex biomacromolecules is a true testament to the power and flexibility of palladium-catalyzed cross-coupling. Furthermore, traditional oligomerization strategies are often not compatible with modified nucleoside derivatives, so the ability to perform cross-coupling directly on oligonucleotides may be the only route to these materials.

Significant progress has been made in the area of palladium-catalyzed coupling of unprotected nucleosides, but there are still challenges left to be conquered. Although many of the classic C–C bond-forming coupling reactions have been demonstrated with unprotected nucleosides, examples of carbon-heteroatom bond formations remain unknown. In contrast, metal-catalyzed carbon-heteroatom coupling reactions of unprotected nucleosides are well precedented [17]. In general, Buchwald-Hartwig-type coupling reactions are less effective in aqueous solvent systems, although recent examples have been reported [123,124,125]. Developing these classes of coupling reactions with unprotected nucleosides would provide access to new classes of nucleoside derivatives. The development of direct coupling reactions of arenes through C–H bond activation represents another attractive area of development. Heterocycles are common substrates for these types of reactions. In addition, the nucleobase heterocycles provide the opportunity for directed C–H functionalization reactions. Finally, further development of direct functionalization of oligonucleotides containing halogenated base residues will provide a route to prepare libraries of oligonucleotides containing modified bases. To date these reactions have been demonstrated with Suzuki and Stille couplings. Extension of these reactions to other classes of cross-coupling reactions would significantly increase the flexibility of these methodologies.
